# A Case of Mortality Caused by *Aeromonas hydrophila* in Wild-Caught Red-Eyed Crocodile Skinks (*Tribolonotus gracilis*)

**DOI:** 10.3390/vetsci7010004

**Published:** 2019-12-29

**Authors:** Jun Kwon, Sang Guen Kim, Sang Wha Kim, Saekil Yun, Hyoun Joong Kim, Sib Sankar Giri, Se Jin Han, Woo Teak Oh, Se Chang Park

**Affiliations:** Laboratory of Aquatic Biomedicine, College of Veterinary Medicine and Research Institute for Veterinary Science, Seoul National University, Seoul 08826, Korea; kjun1002@snu.ac.kr (J.K.); imagine5180@gmail.com (S.G.K.); kasey.kim90@gmail.com (S.W.K.); arseidon@naver.com (S.Y.); hjoong1@nate.com (H.J.K.); giribiotek@gmail.com (S.S.G.); hansj0502@snu.ac.kr (S.J.H.); mike0202@snu.ac.kr (W.T.O.)

**Keywords:** *Aeromonas hydrophila*, bacterial infection, ectotherm, reptile, virulence factor

## Abstract

*Aeromonas hydrophila*, a Gram-negative bacterium commonly found in aquatic environments, is pathogenic to amphibians, reptiles, and mammals. In human medicine, the clinical symptoms of aeromonad infection include not only gastroenteritis but also extraintestinal infections, such as wounds, cellulitis, and septicemia, in immunocompromised and immunocompetent individuals. In this study, ten red-eyed crocodile skinks (*Tribolonotus gracilis*) that shared the same space were found dead 7 days after being shipped from Indonesia. The necropsy revealed *A. hydrophila* to be the causative agent, and the isolates were susceptible to most antibiotics, based on an antimicrobial susceptibility test. Seven virulence factors (*act*, *ast*, *alt*, *aerA*, *fla*, *gcaT*, and *ahyB*) considered to be associated with virulence were detected by PCR. Microscopic examination revealed several necrotic lesions and melano-macrophage centers in the tissue slides. Reptiles caught in the wild for trade experience captivity stress. Furthermore, in the winter, reptiles are easily exposed to the cold atmosphere. These stresses can negatively impact the immunity of these ectotherms, making them vulnerable to *A. hydrophila* infections. Therefore, to avoid such opportunistic infections and mortality following exposure to severe stress, medical care is recommended. The studies of alternatives, such as bacteriophage and bacteriocin, are needed for a preventive application.

## 1. Introduction

*Aeromonas hydrophila* is a Gram-negative bacterium commonly found in aquatic environments [1,2] and is frequently isolated from rivers, lakes, seawater, and animals. Aeromonads are pathogenic to fish, amphibians, reptiles, dogs, and humans. These bacteria are known to cause red-leg syndrome in amphibians and septicemia in many species [3,4]. In human medicine, the clinical symptoms of aeromonad infection include not only gastroenteritis but also extraintestinal infections, such as wounds, cellulitis, and septicemia, in immunocompromised and immunocompetent individuals [2,5,6,7]. The pathogenesis of this bacterium is associated with virulence factors, such as endotoxins, hemolysins, and enolase, and other structural factors, such as the flagella [6,7,8].

Although the infection of Aeromonads is highly probable in tropical reptiles, there is no report of such infection in South Korea. In this study, we describe the mortality of red-eyed crocodile skinks from the perspective of the pathogen.

## 2. Case Description

The red-eyed crocodile skink is a terrestrial lizard that is endemic to the tropical rainforests of New Guinea. *T. gracilis* is a popular species of the genus in the exotic pet trade [9]. An animal importer from Seoul, South Korea, requested necropsies of ten of these lizards that shared the same vivarium and died suddenly 7 days after being shipped from Indonesia. Mortality occurred within 3 days. While 6 of them died in the first 2 days, another 4 were found dead on day 3. The six skinks that died in the first 2 days were preserved frozen at −20 °C until the necropsy, and the other four were kept at 4 °C. The necropsy was performed on the third day, as per the standard protocol.

**Figure 1:**
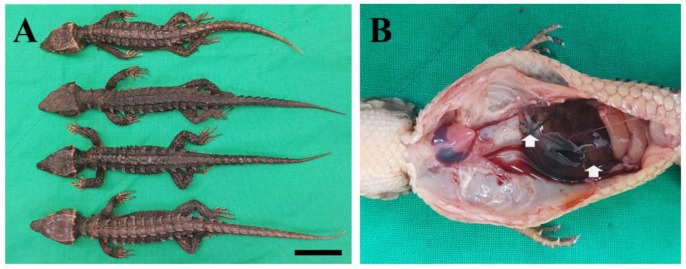
Findings of the examinations. (**A**) Gross examination (bar = 3 cm) and (**B**) hepatic lesions (white arrow) seen at necropsy.

An external examination showed that the average body length of the skinks was 187.5 ± 8.3 mm (except in 2 of them with tails missing). No other remarkable findings were noted. The necropsy revealed hepatic lesions, and 7/10 carcasses had small amounts of clear ascites (Figure 1). Lung and liver tissues were collected and submitted for bacterial culture and DNA extraction. The remaining tissues were fixed in 10% neutral buffered formalin, sectioned at 4 µm, and stained with hematoxylin and eosin for microscopy. The samples were negative for other potential pathogens, such as herpesvirus, mycoplasma, iridovirus, and adenovirus [10,11,12,13,14], based on PCR.

The liver and lung tissues were cultured for bacteria by section imprinting on tryptic soy agar (TSA; Difco, Detroit, MI, USA) for 24 h at 27 °C. All colonies were purified by streaking three times. The 16S rRNA gene was sequenced by MACROGEN (Seoul, Korea) for bacterial identification. The isolates from all organs were positive for *A. hydrophila*, which was confirmed by PCR using *Aeromonas* specific primers (Table 1) [15].

For the detection of virulence and the related genes, PCR was performed for the amplification of 8 virulence factors (*act*, *ast*, *alt*, *aerA*, *gcaT*, *ser*, *ahyB*, and *fla*) (Table 1) [8]. The PCR products were separated by electrophoresis on a 1.5% agarose gel stained with StaySafe^TM^ Nucleic acid gel stain (Real Biotech Corporation, Taiwan). The samples were positive for seven out of the eight factors (*act*, *ast*, *alt*, *aerA*, *fla*, *gcaT*, and *ahyB*), of which four are toxin genes. Cytotoxic enterotoxin (*act*), cytotonic enterotoxin (*ast*, *alt*), and aerolysin (*aerA*) have crucial roles in the onset of infections. The *gcaT* gene is involved in the regulation of extracellular glycerophospholipid-cholesterol acyltransferase [8]. While the flagellin gene (*fla*) is associated with swarming motility, *ahyB* is an elastase gene that is important for the pathogenesis of the organism [16].

Antimicrobial susceptibility tests were performed by disk diffusion. For the disk diffusion method, the bacterial strain was cultured on a Mueller–Hinton agar (BD Difco, Detroit, MI, USA) for 18 h at 35 °C. The test was performed, and the results were analyzed after 18 h incubation according to the protocol of the M45 performance standard for antimicrobial susceptibility testing, third Edition (Clinical Laboratory Standard Institute^TM^, USA). These tests showed that the isolates had intermediate resistance to imipenem and high resistance to vancomycin. They were, however, susceptible to other antimicrobial agents (Table 2).

**Figure 2:**
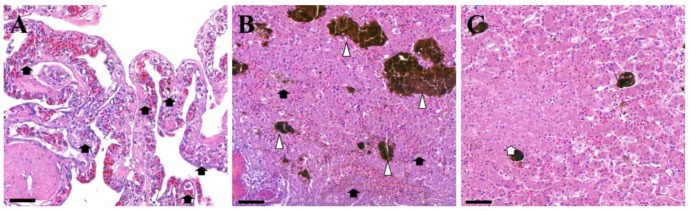
Microscopic examinations. Shown are the microscopic findings in the (**A**) lung and (**B**) liver tissues. HE staining shows the presence of several melano-macrophage centers (arrowhead) and diffused melano-macrophages (black arrow). Necrotic lesion in liver (white arrow, **C**). (bar = 60 µm).

Microscopic examination revealed several necrotic lesions and melano-macrophage centers in the liver tissues (Figure 2). Heterophiles and other inflammatory cells were observed in both the liver and lung tissues.

## 3. Discussion

Aeromonads are common bacteria in a watery environment. This bacterium resides not only in rivers and seawater but also in humid soil. Since the habitual environment of *T. gracilis* is a humid tropical rainforest, they are constantly exposed to aeromonads. Furthermore, *A. hydrophila* can be transferred from these reptiles to humans through touching and biting when they share common space as pets [17]. The existence of virulence factors, such as act, ast, and alt, indicates that these bacteria can cause diseases by oral intake as well. It is thought that aeromonads from animals caught in the wild tend to be susceptible to antibiotic drugs because of their rare exposure to these drugs [18]. This is consistent with our findings that the bacteria are susceptible to most antibiotics.

Reptiles are imported in all seasons. In the winters, in addition to captivity stress, cold temperature-induced stress could also potentially affect the immune systems of these animals. Although the effects of stress on the immune system are complex, previous studies have indicated that besides immune depression, cold stress also induces physiological imbalance by altering the acid-base and ionic balance and lowers the physical functions in ectothermic animals [19,20,21,22,23]. The 10 animals in this study were all imported together in December, when the average temperature in Seoul was −8.3–11.5 °C, according to the Korea Meteorological Administration (http://www.kma.go.kr/eng/index.jsp). It was, therefore, cold enough to induce cold stress in these reptiles. Although *A. hydrophila* was the cause of the mortality, stress associated with captivity, transfer, and low temperature could have contributed to the pathogenesis. Therefore, to avoid such opportunistic infections and mortality following exposure to severe stress, medical care is recommended when any clinical signs are observed. The studies of alternatives, such as bacteriophage and bacteriocin, are needed for preventive application.

## 4. Conclusions

In this case, mortality due to *A. hydrophila* septicemia in reptiles was investigated. *A. hydrophila* is a common pathogen, but this is the first report of *Aeromonas* infection in reptiles in South Korea. The isolates contained several virulence factors that are considered to be important factors in human infection. Although these isolates had susceptibility towards a number of antibiotics, we need to consider alternatives in the case of antimicrobial resistance.

## Figures and Tables

**Table 1:** Primers used in this study.

Primers	Sequences	Target Gene
act	5′-AGAAGGTGACCACCACCAAGAACA-3′	cytotoxic enterotoxin
	5′-AACTGACATCGGCCTTGAACT C-3′	
ast	5′-TCTCCATGCTTCCCTTCCACT-3′	cytotonic enterotoxin
	5′-GTGTAGGGATTGAAGAAGCCG-3′	
fla	5′-TCCAACCGTYTGACC TC-3′	flagellin
	5′-GMYTGGTTGCGRATG GT-3′	
gcaT	5′-CTCCTGGAATCCCAAGTATCA G-3′	glycerophospholipid: cholesterol acyltranferase
	5′-GGCAGGTTGAACAGCAGTATC T-3′	
ser	5′-CACCGAAGTATTGGGTCAGG-3′	serine protease
	5′-GGCTCATGCGTAACTCTGGT-3′	
ahyB	5′-ACACGGTCAAGGAGATCAAC-3′	elastase
	5′-CGCTGGTGTTGGCCAGCAGG-3′	
alt	5′-TGACCCAGTCCTGGCACGGC-3′	cytotonic enterotoxin
	5′-GGTGATCGATCACCACCAGC-3′	
aerA	5′-CCTATGGCCTGAGCGAGAAG-3′	aerolysin
	5′-CCAGTTCCAGTCCCACCACT-3′	
*Aeromonas* species specific primers		
A. hyd	5′-AGTCTGCCGCCAGTGGC-3′	gyrB
	5′-CRCCCATCGCCTGTTCG-3′	
A. 16S	5′-CGACGATCCCTAGCTGGTCT-3′	16S rRNA gene
	5′-GCCTTCGCCACCGGTAT-3′	

**Table 2:** Susceptibility of *A. hydrophila* isolates from carcasses.

Antibiotics	*A.hydrophila* Isolates			
	1	2	3	4
IPM	I	I	I	I
MEM	S	S	S	I
VAN	R	R	R	R
AZM	S	S	S	S
CHL	S	S	S	S
GEN	S	S	S	S
TET	S	S	S	S
ATM	S	S	S	S
AMK	S	S	S	S
CRO	S	S	S	S
FEP	S	S	S	S
CAZ	S	S	S	S
SXT	S	S	S	S
TZP	S	S	S	S
LVX	S	S	S	S
CIP	S	S	S	S

IPM; imipenem, MEM; meropenem, VAN; vancomycin, AZM; azithromycin, CHL; chloramphenicol, GEN; gentamycin, TET; tetracycline, ATM; aztreonam, AMK; amikacin, CRO; ceftriaxone, FEP; cefepime, SXT; trimethoprim-sulfamethoxazole, TZP; piperacillin-tazobactam, LVX; levofloxacin, CIP; ciprofloxacin, I; intermediate, S; susceptible, R; resistant.
